# Rare Site of Ectopic Pregnancy in a Patient with Bicornuate Uterus

**DOI:** 10.1155/2016/6395154

**Published:** 2016-11-30

**Authors:** Ayman Shehata, Mohamed El Namoury, Mostafa Heider

**Affiliations:** Tanta University, Tanta, Egypt

## Abstract

Ectopic pregnancy occurs in about 1-2% in normal cycles but in IVF cycles the rate jumps to 4%. No definite cause for ectopic pregnancy was detected, but many risk factors were described as abnormal tube, pelvic infection, or surgery. In this case report we found 2 abnormalities in eight-year infertile woman; the first abnormality was bicornuate uterus and the second abnormality was the site of ectopic pregnancy which was in between the two horns of uterus. ‎This is the only case reported with primary abdominal pregnancy with bicornuate uterus and both healthy ovaries and tubes.‎ The case was unstable and managed by laparotomy and repair of ectopic site after enucleation of sac using Vicryl 2/0. The case was discharged 24 hours after operation in good health.

## 1. Introduction

Ectopic pregnancy was firstly described by Albucasis in the 11th century [1]. The rate of ectopic pregnancy is about 1-2% in spontaneous pregnancy and as high as 4% among those using assisted reproductive technology [2]. Ectopic pregnancy accounts for 10% of maternal deaths in the first trimester so it must be considered the most important cause of death during the first trimester [3].

Salpingitis of any etiology causing partial or complete tubal block, pelvic adhesions impeding tubal motility, tubal abnormalities in shape or ciliary movement, intrauterine device, and previous ectopic pregnancy are the well known risk factors for ectopic pregnancy [2].

The commonest site for ectopic pregnancy is the fallopian tube representing 95% with all its parts (intramural, ischemic, and ampullary parts and infundibulum). In rare cases of ectopic pregnancy (5%), the site may be a matter of case reports denoting that ectopic pregnancy may be corneal, rudimentary horn pregnancy [4], ovarian, abdominal [5], cervical, heterotopic [6], or of unknown location [2, 7, 8].

Management depends on hemodynamic stability, age, b-HCG level, and cardiac pulsation. Many modalities of ectopic pregnancy included medical treatment with methotrexate injection [9], laparoscopic management, and laparotomy with repair or removal of the affected fallopian tube [10].

## 2. Case Presentation

A patient aged 27 years was admitted to Department of Obstetrics and Gynecology, Tanta University Hospitals, in a shock state with a pulse rate of 120 b/m and blood pressure of 80/50.

The patient had history of primary infertility for 6 years and history of ovulation induction in the cycle preceding this pregnancy, history of uterine anomaly, and history of amenorrhea for 7 weeks. This means that this pregnancy was induced by ovulation induction.

**Figure 1:**
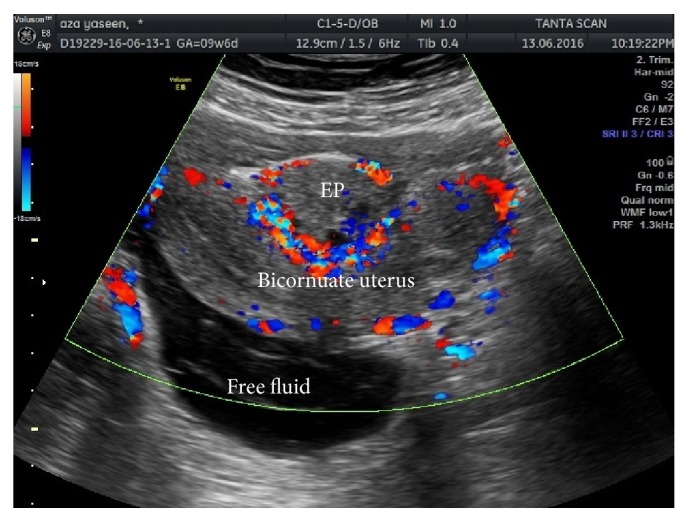
Doppler ultrasound showing bicornuate uterus and pelvic free fluid with the ectopic pregnancy site showing “Ring of Fire.” EP: ectopic pregnancy.

Immediate routine investigations included complete blood count and renal and liver functions with bleeding and coagulation times. All investigations were within normal except for hemoglobin which was 9.7 g/dl and hematocrit value of 30.21. Specific investigations requested were pregnancy test and b-HCG level which revealed pregnancy with b-HCG level of 2140 IU. Immediate resuscitation was done by insertion of 2 wide pore cannulas and intravenous fluids with immediate ultrasound which revealed bicornuate uterus and moderate free fluid in the abdomen; pelvic hematoma about 10 × 3 cm was also detected in the pelvis. The uterus was empty and pregnancy sac was found between the two horns of bicornuate uterus with high peripheral vascularity surrounding the sac “Ring of Fire,” Figure 1.

Taping of free fluid which revealed altered dark blood confirmed the diagnosis of ectopic pregnancy. The patient did not receive preoperative blood and was immediately transferred to operation room with 2 units of crossmatched blood ready to be used intraoperatively or postoperatively. Laparotomy revealed moderate to severe internal hemorrhage with blood clots in pelvis. Pelvic hematoma was anterior to the bicornuate uterus between uterus and bladder covering pregnancy sac. Both fallopian tubes and ovaries were inspected and were healthy with no lesions denoting that the ectopic pregnancy was primary.

**Figure 2:**
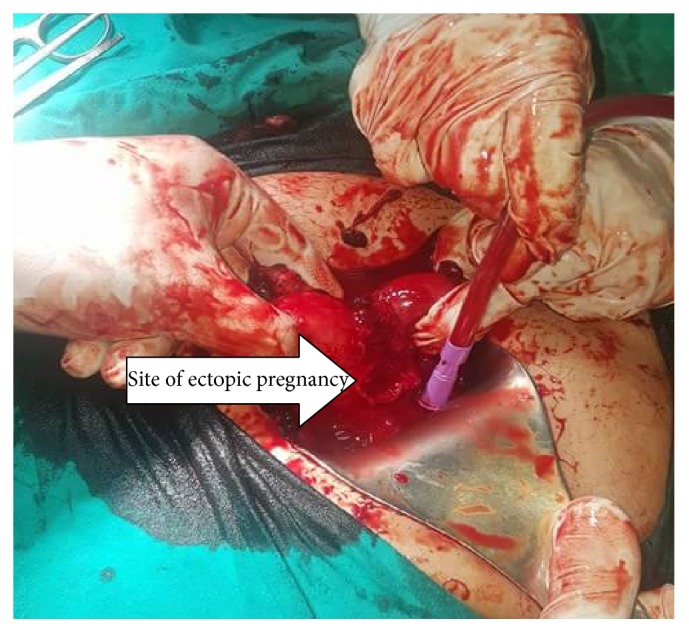
Laparotomy with bicornuate uterus and site of ectopic pregnancy arrow.

Suction of blood and clearing retrovesical hematoma revealed pregnancy sac in between the 2 horns of bicornuate uterus which was enucleated and debridement of its site and repair of its bed to control bleeding as shown in Figure 2.

**Figure 3:**
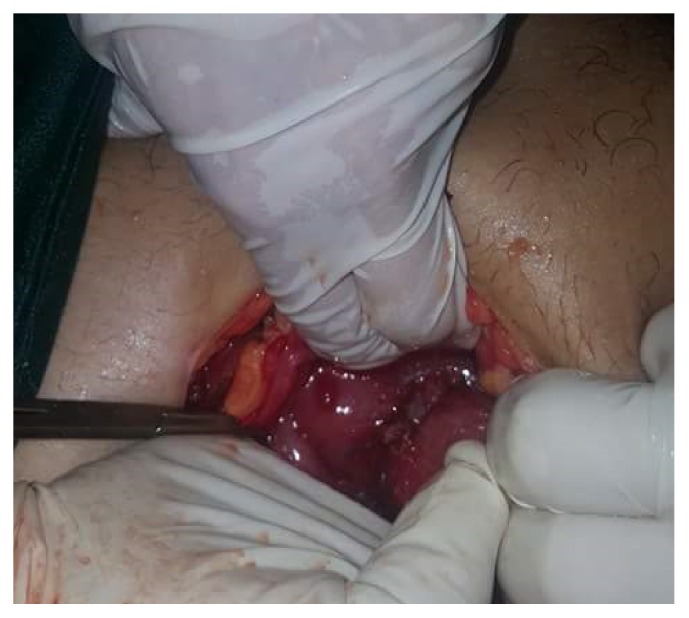
Repair of site of ectopic pregnancy and suction of blood.

Closure of ectopic pregnancy site by Vicryl 2/0 and the end picture were shown in Figure 3.

The abdomen was irrigated by 2 liters of warm saline and closure of abdominal wall was then commenced in anatomical layers. Postoperative follow-up consisted of monitoring vital signs and urine output. The patient was given the 2 units of blood one inside operation room and one after 12 hours. Complete blood count was requested before discharge with hemoglobin 10.8 g/dl. The case was discharged 24 hours after operation in a good condition.

## 3. Discussion

Abdominal pregnancy is a rare type of ectopic pregnancy occurring in 1 in 8000 pregnancies. Although rare, abdominal pregnancy has high maternal mortality compared to other types of ectopic pregnancies [11].

Abdominal pregnancy has 2 types, secondary abdominal pregnancy which is the commonest type and occurs due to reimplantation after rupture of its original site in the tube or less commonly the ovary. The primary abdominal pregnancy occurs when implantation occurs directly in the peritoneum, saving the tubes and ovaries. The primary type is very rare; only 24 cases had been reported by 2007 [12].

The sites reported for abdominal ectopic pregnancy were Douglas pouch, omentum, intestine and its mesentery, mesosalpinx, spleen, liver, and diaphragm [13].

In this case we represent a rare site for primary abdominal pregnancy where the uterus is bicornuate and both tubes and ovaries were healthy. The sac was implanted on the peritoneum between the 2 horns of uterus. This is the only case in literature of primary abdominal pregnancy where the uterus is ‎bicornuate and both ovaries and tubes are healthy.‎

The question here is, does malformed uterus increases the chance of ectopic pregnancy or has an effect on the site of implantation?
